# Role of adenomatous polyposis coli in proliferation and differentiation of colon epithelial cells in organoid culture

**DOI:** 10.1038/s41598-021-83590-6

**Published:** 2021-02-17

**Authors:** Daisuke Yamazaki, Osamu Hashizume, Shiho Taniguchi, Yosuke Funato, Hiroaki Miki

**Affiliations:** Department of Cellular Regulation, Research Institute for Microbial Diseases, Osaka University, Suita, Osaka 565-0871 Japan

**Keywords:** Cancer, Cell biology

## Abstract

Adenomatous polyposis coli (APC) is a tumor-suppressing protein whose inactivation triggers the formation of colorectal polyps. Numerous studies using cell lines or genetically engineered mice have revealed its role in suppressing Wnt/β-catenin signaling pathway and regulating cell proliferation and differentiation. Here, we performed genetic analyses of APC using a three-dimensional organoid culture of mouse colon epithelia, which enables the detailed examination of epithelial properties. Analyses of *Apc*-knockout colon organoids not only confirmed the importance of APC in suppressing Wnt/β-catenin signaling and regulating cell differentiation, but also revealed several novel features: a significant decrease in proliferating speed and an increase in cross-sectional area of cells. Moreover, we found a significant number of lysozyme-positive Paneth-like cells, which were never observed in wild-type colon tissues or organoids, but have been reported to emerge in colon cancers. Therefore, APC autonomously suppresses ectopic differentiation into lysozyme-positive cells, specifically in the colon epithelia. Colon organoids would be an ideal material to investigate the molecular mechanism and biological importance of the ectopic differentiation associated with cancer development.

## Introduction

*Adenomatous polyposis coli* (*Apc*) is a tumor suppressor, whose mutation is responsible for the development of familial adenomatous polyposis (FAP), an autosomal dominant disorder characterized by multiple colorectal polyps^1,2^. This gene is also very frequently (> 80%) mutated in sporadic colon cancers^3,4^. Its product, APC, is a giant protein with multiple functions; its most characterized role is the suppression of Wnt/β-catenin signaling^5,6^. Wnt/β-catenin signaling pathway is triggered by extracellular Wnt proteins that bind to their receptors, Frizzled, on the plasma membrane of target cells. This ligand-receptor interaction inactivates the β-catenin destruction complex, composed of APC, Axin, and GSK3β^6^. β-catenin then escapes from cytosolic degradation and accumulated β-catenin enters the nucleus, where it associates with the transcription factor TCF/LEF and stimulates the expression of various target genes. Therefore, mutational inactivation of APC abrogates the β-catenin destruction complex and results in aberrant activation of Wnt/β-catenin signaling, which is considered as the primary step in colon cancer development.

To date, many genetic analyses using mice have confirmed the critical importance of APC in cancer development in the intestine. Forward genetics using chemical mutagenesis resulted in the isolation of the first FAP mouse model strain, which developed multiple intestinal adenomas at an early age and had an inactivating mutation in one *Apc* allele (*Apc*^Min/+^ mice)^7,8^. Moreover, multiple reverse genetics by targeted deletion of functionally important but different exons of *Apc* showed that their heterozygotes also spontaneously formed similar intestinal adenomas^9–11^, thereby confirming the important role of APC in suppressing colon cancer development, as supposed from the symptoms of FAP patients.

Intestinal cancers originate from epithelial cells in the mucosa. Sato et al.^12^ developed an organoid culture system for intestinal epithelia using three-dimensional (3D) matrixes supplemented with various stimulants, which can maintain the in vivo characteristics of intestinal epithelia containing both stem and differentiated cells. The role of APC has been studied using this culture system and *Apc*-knockout (KO) and -knockdown (KD) organoids were generated using epithelial cells derived from the small intestine^13–16^. These studies showed that APC inactivation enables the culture to proceed without supplementation with R-spondin1^13–15^, a Wnt/β-catenin signaling stimulant normally required for the organoid culture, and suppresses cell differentiation^16^. These organoid culture results, which are completely free from other types of cells that exist close to the epithelia in vivo, are consistent with previously reported functions of APC, but clearly establish the direct importance of APC in the intestinal epithelia. However, it should be noted that these analyses were performed using the small intestine-derived organoids, and not the colon-derived ones. Considering the qualitative differences between them, such as the requirement of Wnt ligands in the culture supplements^13^, functional analyses of APC using the colon-derived organoids have been awaited.

In this study, we generated *Apc*-KO organoids of the colon epithelia using the CRISPR/Cas9 system. As expected, *Apc*-KO suppressed cell differentiation and maintained proliferation, even in media without stimulants. Also, we found that cross-sectional cell area of cells was significantly larger in *Apc*-KO organoids. Furthermore, we discovered that *Apc*-KO promoted the ectopic differentiation of lysozyme-positive cells, which do not normally exist in the colonic epithelia, suggesting a novel and unique role of APC in regulating cell differentiation.

## Results

### Disruption of *Apc* in epithelial organoids derived from mouse colon

**Figure 1:**
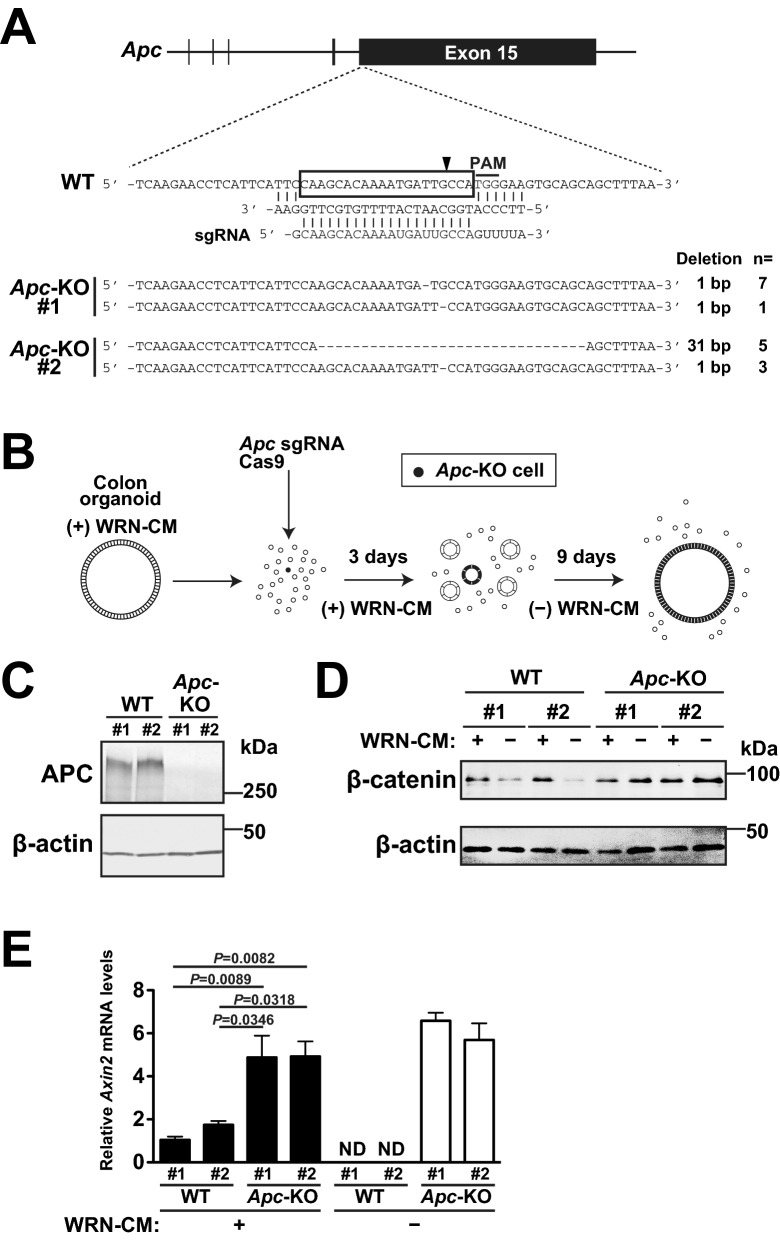
Disruption of *Apc* in epithelial organoids derived from the mouse colon. (**A**) Schematic representation of the targeted exon of mouse *Apc* locus and the nucleotide sequences of WT and KO alleles from two independent organoid cell clones (*Apc*-KO#1 and *Apc*-KO#2). The number of deleted nucleotides in each sequence and the number of times each sequence was detected during verification are also shown. The protospacer is boxed and protospacer adjacent motif (PAM) is overlined. The arrowhead indicates the cleavage site. (**B**) Strategy to generate *Apc*-KO organoid cell clones. Colon organoids cultured in the presence of WRN-CM were dissociated with trypsin to obtain single-cell suspensions. sgRNA and Cas9 encoding plasmids were transfected into the cells. Transfected cells suspended in Matrigel were cultured in the presence of WRN-CM for 3 days, and then *Apc*-KO organoid cell clones (thick black) were selected by WRN-CM removal. (**C**) Immunoblot analysis of WT and *Apc*-KO organoids with the indicated antibodies. (**D**) WT and *Apc*-KO organoids were cultured in the presence or absence of WRN-CM for 3 days. Lysates of organoids were immunoblotted with the indicated antibodies. Full-length blots of (**C**) and (**D**) are presented in Supplementary Fig. S2. (**E**) qRT-PCR for *Axin2* in WT and *Apc*-KO organoids cultured in the presence or absence of WRN-CM for 3 days. Expression normalized to *Gapdh*. Data are expressed as mean ± s.e.m. (n = 3). ND, not detected. Tukey’s multiple comparison test was used after one-way analysis of variance to calculate *P* values.

We physically isolated crypts from the mouse colon and cultured them in 3D Matrigel, according to a previously reported method^17^. To maintain stem cell fractions, we supplemented conditioned medium (CM) obtained from the supernatants of the culture of L cells that express Wnt3a, R-spondin3, and Noggin (WRN)^18,19^. *Apc* disruption was performed using the CRISPR/Cas9 system by targeting the sequence in the last exon (exon 15), which is schematically illustrated in Fig. 1A. The targeting sequence is located at the beginning of exon 15, and most part of exon 15 is expected to be inactivated by gene mutations. Importantly, deletion of whole exon 15 in mice increases the level of β-catenin expression and causes tumorigenesis in colon epithelia^20^. We introduced the sgRNA-expressing constructs into single cells isolated from the organoid culture using plasmid transfection. Three days after transfection, WRN-CM was removed to select *Apc*-deficient organoids, and cells were cultured for an additional 9 days (Fig. 1B). We obtained two independent single cell-derived organoid cell clones, *Apc*-KO#1 and *Apc*-KO#2, which had frameshift mutations in and around the original target sequence (Fig. 1A and Supplementary Fig. S1). Both *Apc*-KO organoids lacked APC expression (Fig. 1C) and were used for subsequent analyses. As exon 15 encodes functionally important domains to suppress Wnt/β-catenin signaling^5^, we examined the activation status of Wnt/β-catenin signaling. Two independent wild-type (WT) and *Apc*-KO organoids were cultured in the presence or absence of WRN-CM for 3 days and subjected to β-catenin immunoblotting analyses. Removal of WRN-CM significantly reduced β-catenin levels in WT organoids but did not affect β-catenin levels in *Apc*-KO organoids (Fig. 1D). In addition, we performed quantitative reverse transcription polymerase chain reaction (qRT-PCR) analyses for *Axin2*, the most characterized target gene for Wnt/β-catenin signaling in intestinal epithelial cells^13,15,21^. The results showed constitutive elevation of *Axin2* mRNA levels in *Apc*-KO organoids (Fig. 1E). Collectively, Wnt/β-catenin signaling is constitutively activated in *Apc*-KO organoids, which is consistent with the major role of APC in suppressing Wnt/β-catenin signaling. In addition, *Axin2* expression was much higher in *Apc*-KO organoids than in WT organoids even in the presence of WRN-CM as described previously^15^. Considering these phenotypes shown in Figs. 1C–E were common to two independent *Apc*-KO organoid clones, it is likely that constitutive activation of Wnt/β-catenin signaling is caused by gene edition of *Apc* in these *Apc*-KO organoids.

### Growing properties of *Apc*-KO organoids

**Figure 2:**
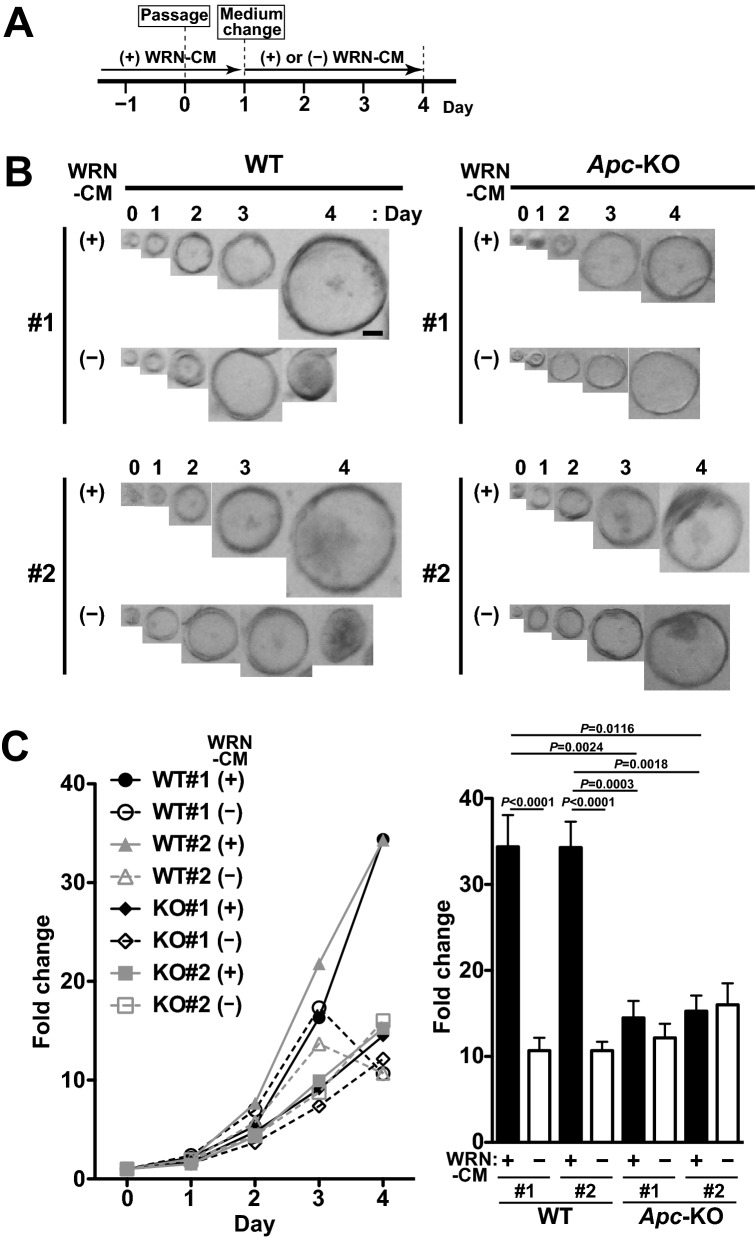
Growing properties of *Apc*-KO organoids. (**A**) Experimental schedule. Colonic organoids were passaged on Day 0, and then cultured in the presence of WRN-CM to facilitate organoid formation. The medium was changed to the fresh medium supplemented with (+) or without (−) WRN-CM on Day 1, and then the organoids were cultured for 3 more days (Days 2–4). (**B**) Time course of development of an organoid. Scale bar, 50 μm. (**C**) The growth rate of organoids was determined by measuring the projected area of the organoid normalized to the initial size. The growth rate of each organoid on Day 4 is also plotted (right). Data are expressed as mean ± s.e.m. (n = 24 organoids for each group). Dunn’s multiple comparison test was used after Kruskal–Wallis test to calculate *P* values.

We then monitored the growth of each organoid during the 4-day culture after passage and the medium change on Day 1 (Fig. 2A). In the presence of WRN-CM, WT organoids continued to grow, maintaining a smooth and spherical morphology (Fig. 2B). However, they stopped growing on Day 3 and became smaller and darker on Day 4 in the absence of WRN-CM, probably reflecting the accumulation of dead cells or their debris inside them. In contrast, *Apc*-KO organoids continued to grow, irrespective of the presence or absence of WRN-CM. To quantify the results, the size of each organoid was measured and plotted (Fig. 2C), confirming the dependency of WT organoids on WRN-CM. This quantification also revealed that the growth rate of *Apc*-KO organoids was significantly lower than that of WT organoids in the presence of WRN-CM, suggesting an inhibitory role of APC in the growth of colon epithelial cells in organoid culture.

**Figure 3:**
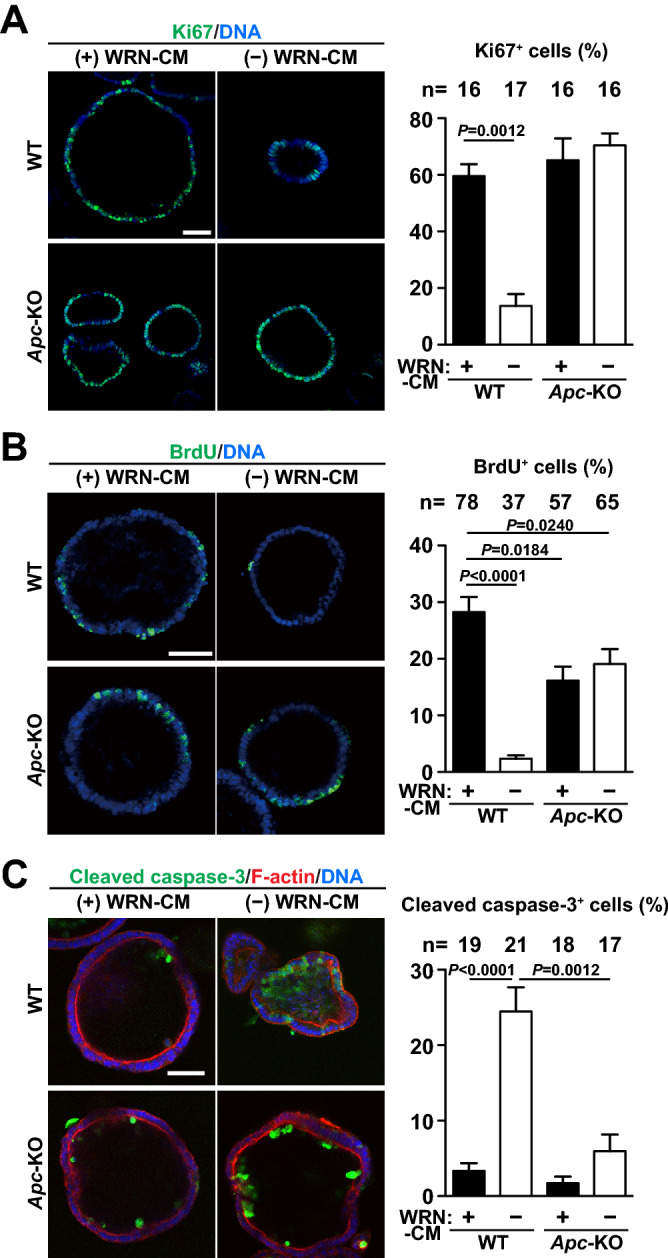
Cell proliferation and apoptosis in *Apc*-KO organoids. Organoids formed in the presence of WRN-CM were cultured in the presence or absence of WRN-CM for 3 more days. (**A**, **C**) The organoids were fixed and then stained using anti-Ki67 antibody (**A**, green) and anti-cleaved Caspase-3 antibody (**C**, green). Counter stain, DAPI for DNA (blue) and phalloidin for F-actin (**C**, red). (**B**) After labeling with BrdU for 8 h, the organoids were fixed and stained with anti-BrdU antibody (green) and DAPI (blue). We counted the number of Ki67-positive (**A**), BrdU-positive (**B**), cleaved caspase-3-positive (**C**) and DAPI-positive (**A–C**) cells in the largest confocal section for each organoid. The ratio of the number of positive cells for each marker to that of the nuclei in the same section was quantified for each group. The total number of organoids analyzed is shown above each graph. Data are expressed as mean ± s.e.m. Dunn’s multiple comparison test was used after Kruskal–Wallis test to calculate *P* values. Scale bar, 50 μm.

To investigate the proliferation status of the cells in the organoid culture, we fixed and stained them for Ki67, a stem/progenitor cells marker with proliferating potential^22^. The results indicated that more than half of the cells were positive in WT organoids cultured in the presence of WRN-CM, but the positive rate greatly decreased by its removal (Fig. 3A). In contrast, *Apc*-KO organoids maintained a high rate of positive cells, irrespective of the presence or absence of WRN-CM. We also performed 5-Bromo-2′-deoxyuridine (BrdU) incorporation assays to directly label the cells when they replicate their DNA and found a drastic decrease in the number of positive cells in WT organoids by depleting WRN-CM (Fig. 3B), as seen in the Ki67-staining experiments. Moreover, the rate of DNA-replicating cells was moderately decreased in *Apc*-KO organoids compared to WT organoids, which probably contributed to the moderate decrease in the growing speed of *Apc*-KO organoids as observed in Fig. 2C.

Organoid growth is not only affected by cell proliferation, but also by cell death. Therefore, we stained organoids with an antibody that specifically recognizes activated caspase 3 (cleaved caspase 3) for visualizing apoptotic cells. A few positive cells were observed mostly at the inner side of both *Apc*-KO and WT organoids in the presence of WRN-CM (Fig. 3C). However, removal of WRN-CM induced massive apoptosis only in WT organoids. Therefore, cell survival, as well as cell proliferation, of WT organoids is also critically dependent on stimulation with WRN-CM.

### Increase in cross-sectional area of cells in *Apc*-KO organoids

**Figure 4:**
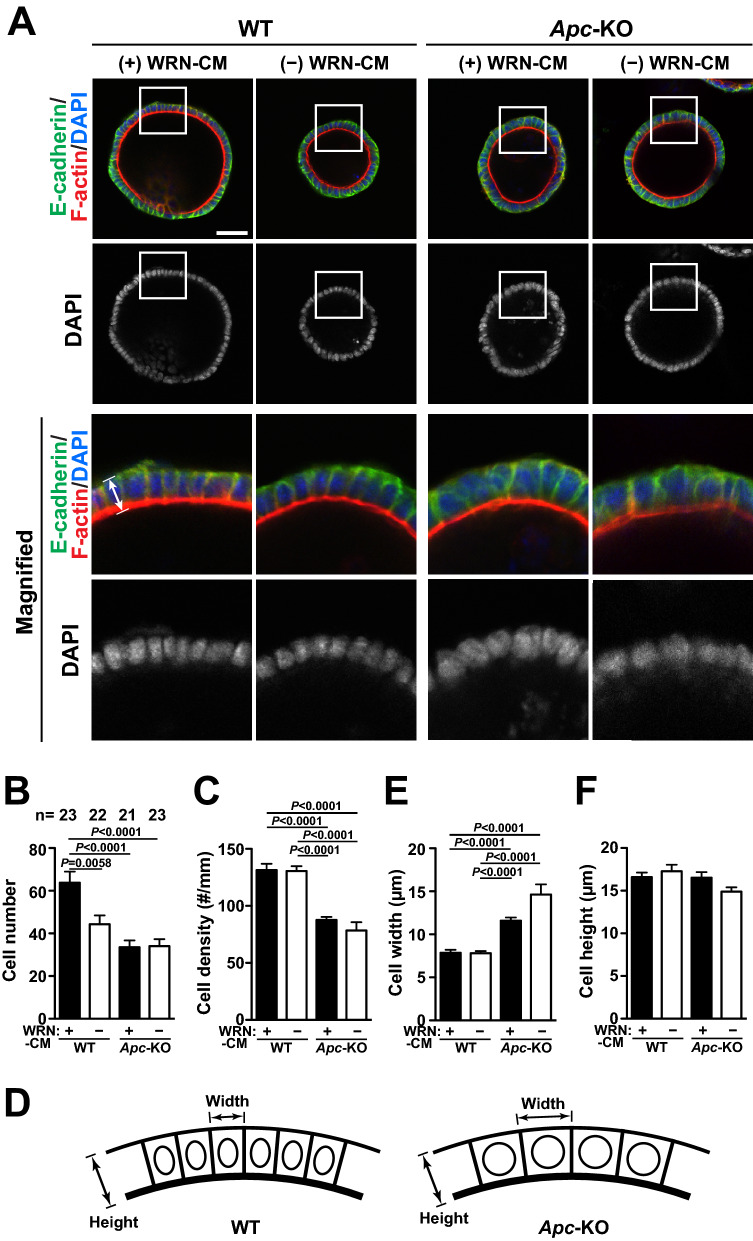
Increase in cross-sectional area of cells in *Apc*-KO organoids. Organoids formed in the presence of WRN-CM were cultured in the presence or absence of WRN-CM for 3 more days. (**A**) The organoids were stained with anti-E-cadherin antibody for cell–cell adhesion, phalloidin for F-actin, and DAPI for DNA. Magnified images of the boxed areas are also shown. Scale bar, 50 μm. (**B**) Cell number was measured by counting DAPI-positive nuclei in the largest confocal section for each organoid. The total number of organoids analyzed is shown above the graph. Data are expressed as mean ± s.e.m. Bonferroni’s multiple comparison test was used after one-way analysis of variance to calculate *P* values. (**C**) Cell density was calculated by dividing the cell number by the length of the perimeter in the same section. Data are expressed as mean ± s.e.m. (n = 19 organoids for each group). Dunn’s multiple comparison test was used after Kruskal–Wallis test to calculate *P* values. (**D**) Cross-sections of WT and *Apc*-KO organoids are illustrated schematically. Cell width and cell height are indicated by double headed arrows. (**E**) Cell width (depicted in **D**) was calculated by taking the reciprocal of cell density shown in **C**. Data are expressed as mean ± s.e.m. (n = 19 organoids for each group). Dunn’s multiple comparison test was used after Kruskal–Wallis test to calculate *P* values. (**F**) Cell height (indicated by double-headed arrow in **A**) was measured in the largest confocal section for each organoid. Data are expressed as mean ± s.e.m. (n = 20 organoids for each group).

To investigate the status of cells in the organoid culture in more detail, we stained the organoids with anti-E-cadherin antibody, DAPI, and phalloidin to visualize cell–cell adhesion, DNA, and actin filaments (F-actin), respectively (Fig. 4A). We first measured the number of cells by counting the number of the nuclei in the largest confocal section for each organoid. The quantified results showed that the cell number in WT organoids was larger when they were cultured in the presence of WRN-CM (Fig. 4B). We also found that the number of cells in *Apc*-KO organoids was significantly lower than that in WT organoids in the presence of WRN-CM. As shown in Fig. 2C, the size of the organoids differed depending on the *Apc* status and culture conditions. Therefore, we determined the cell density by dividing the cell number by the length of the perimeter of the same confocal section (Fig. 4C). The results showed that cell density was not affected by WRN-CM, and more importantly, it was significantly decreased in *Apc*-KO organoids compared to WT organoids. A decrease in cell density in organoids indicates an increase in the width of each cell, which is depicted in Fig. 4D. Indeed, when cell width was calculated by taking the reciprocal of cell density, it was significantly wider in *Apc*-KO organoids than in WT organoids (Fig. 4E). By contrast, the height of cells from the basement line in the confocal image, which was visualized using F-actin staining (schematically shown in Fig. 4A), was not significantly different between WT and *Apc*-KO organoids (Fig. 4F). Taken together, these results suggest that the cross-sectional area of *Apc*-KO cells is larger than that of WT cells.

### Suppression of cell differentiation in *Apc*-KO organoids

**Figure 5:**
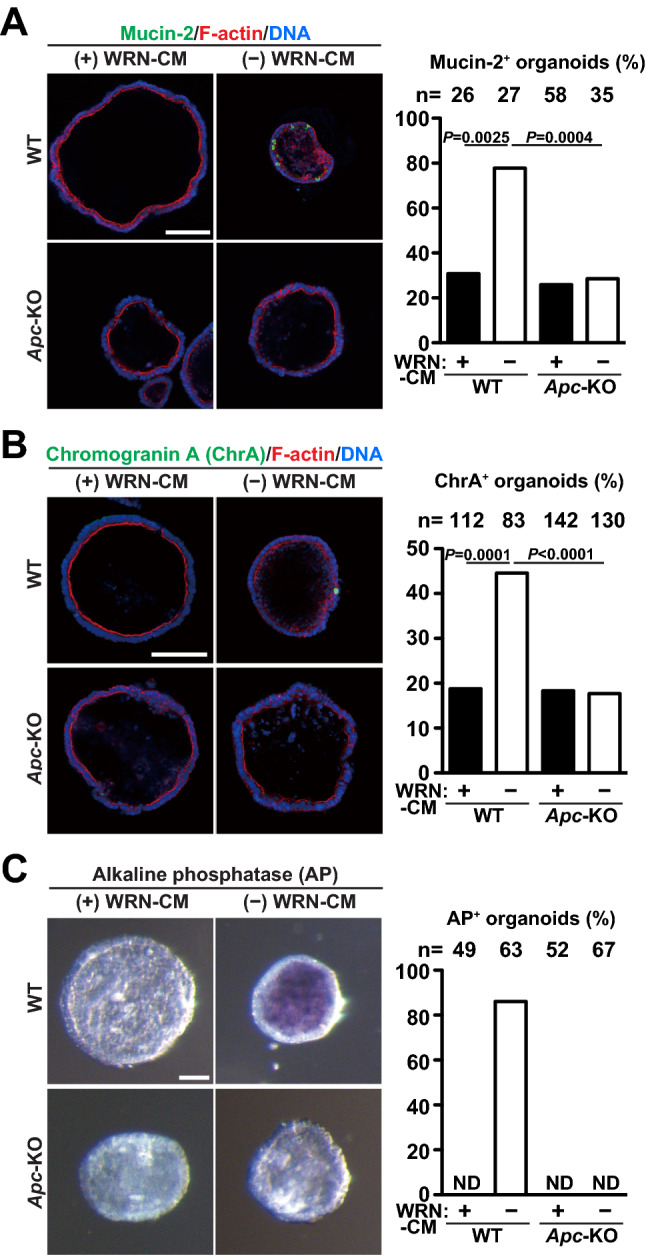
Suppression of cell differentiation in *Apc*-KO organoids. Organoids formed in the presence of WRN-CM were cultured in the presence or absence of WRN-CM for 3 more days. (**A**, **B**) The organoids were stained with anti-Mucin-2 antibody (**A**, green) and anti-chromogranin A antibody (**B**, green). Counter stain, phalloidin for F-actin (red) and DAPI for DNA (blue). The ratio of organoids containing at least one Mucin-2-positive (**A**) and chromogranin A-positive (**B**) cells to total organoids was quantified for each group. The total number of organoids analyzed is shown above each bar. Dunn’s multiple comparison test was used after Kruskal–Wallis test to calculate *P* values. (**C**) Alkaline phosphatase activity (stained purple) was detected in the organoids. The ratio of organoids containing at least one alkaline phosphatase-positive cell to total organoids was quantified for each group. The total number of organoids analyzed is shown above each bar. *ND* not detected. Scale bar, 100 μm.

Having characterized the growing properties of *Apc*-KO colon organoids, we next examined their differentiation capacity. Colonic epithelia are known to be composed of various types of differentiated cells, including goblet cells, enteroendocrine cells, and absorptive cells. WRN-CM, which is used as a supplement for maintaining the organoid culture in our experiments, augments stem cell properties and suppresses cell differentiation^18^. Indeed, when we subjected WT organoids to immunofluorescence-staining or enzymatic activity-staining analyses for visualizing the differentiated cells (goblet cells: Mucin-2, enteroendocrine cells: chromogranin A, and absorptive cells: alkaline phosphatase), we observed only a tiny fraction of organoids with positively stained cells (Fig. 5A–C). Therefore, we removed WRN-CM from the medium and found a significant increase in the number of positive organoids, confirming that WT organoids maintained their differentiation capacity. In contrast, the positive rate of *Apc*-KO organoids for differentiated cells remained low and was not affected by WRN-CM removal. These results indicate that *Apc*-KO suppresses the differentiation of colon epithelia, which is consistent with its proliferating nature in the absence of WRN-CM (Figs. 2 and 3).

To further investigate the mechanistic details, cell differentiation was potently induced by two established methods using chemicals. The treatment with L-161982, an inhibitor for prostaglandin receptor EP4, promoted production of absorptive cells in WT organoids^23,24^, but not in *Apc*-KO organoids (Supplementary Fig. S3A). L-161982 is supposed to promote degradation of β-catenin through β-catenin destruction complex^24^, and thus, it cannot induce differentiation in *Apc*-KO organoids lacking APC, an essential component of this complex^5^. Next, we treated organoids with the γ-secretase inhibitor, DBZ, which inhibits Notch activation and causes overproduction of Goblet cells in intestinal epithelial cells^13,25^. In contrast to L-161982, DBZ treatment increased the number of Mucin2-expressing cells in *Apc*-KO organoids as well as in WT organoids (Supplementary Fig. S3B). Thus, *Apc*-KO has no effect on cell differentiation induced by inhibition of Notch signaling in colon organoids.

### Ectopic emergence of lysozyme-positive cells in *Apc*-KO organoids

**Figure 6:**
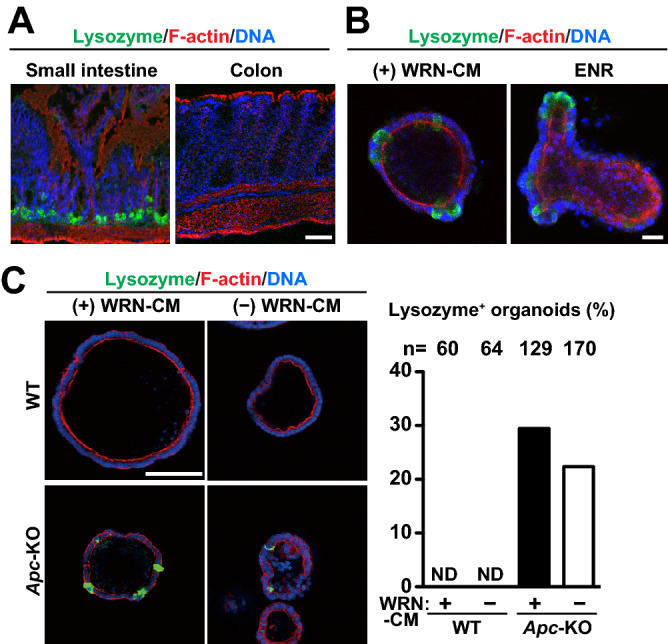
Ectopic emergence of lysozyme-positive cells in *Apc*-KO organoids. (**A**) Sections of the small intestine and colon dissected from WT mice were subjected to immunofluorescence staining for lysozyme (green). Counter stain, phalloidin for F-actin (red) and DAPI for DNA (blue). Scale bar, 50 μm. (**B**) Organoids derived from the small intestine were cultured in the presence of WRN-CM (left) or in ENR medium (right). ENR medium contains EGF, noggin and R-spondin1-CM but not Wnt (see Methods). The organoids were stained with anti-lysozyme antibody (green). Scale bar, 20 μm. (**C**) Colon organoids formed in the presence of WRN-CM were cultured in the presence or absence of WRN-CM for 3 more days. They were stained with anti-lysozyme antibody (green). The ratio of organoids containing at least one lysozyme-positive cell to total organoids was quantified for each group. The total number of organoids analyzed is shown above each bar. *ND* not detected. Scale bar, 100 μm.

Paneth cells, which maintain the stem cell niche by secreting trophic factors at the bottom of the crypts in the small intestine^26–28^, do not normally exist in the colon. Indeed, when tissue sections obtained from the small intestine and colon were stained for lysozyme, a maker for Paneth cells^29^, clear positive signals were observed in the small intestine, but not in the colon (Fig. 6A). However, it has been reported that such lysozyme-positive cells ectopically emerge in human colon cancer tissues and in mouse colon lacking *Apc* genes^30–32^. Therefore, we subjected the organoids, which are composed of only epithelial cells, to lysozyme staining. As expected, the organoids derived from the small intestine possessed many lysozyme-positive cells (Fig. 6B). In contrast, WT colon organoids had no lysozyme-positive cells even when WRN-CM was removed from the medium to induce cell differentiation (Fig. 6C), implying that the lineage difference between the small intestine and the colon is still maintained in the organoid culture. However, we observed a significant number of lysozyme-positive cells in *Apc*-KO organoids, irrespective of the presence or absence of WRN-CM. Therefore, contrary to the case of the normal differentiation, *Apc*-KO rather autonomously promotes the differentiation of lysozyme-positive cells, without the help of other types of cells.

## Discussion

In this study, we generated *Apc*-KO organoids using colon epithelium-derived cells and analyzed their proliferation, morphology, and differentiation capacity. The analyses of colon *Apc*-KO organoids showed constitutive activation of Wnt/β-catenin signaling and suppression of differentiation into goblet cells, enteroendocrine cells, and absorptive cells. These results are consistent with those of previous studies on *Apc*-KO and -KD organoids derived from the small intestine^13,15,16^, indicating the common and general functions of APC in the intestine. However, we also noticed several different features specific to *Apc*-KO organoids, which have not been reported so far.

We found that *Apc*-KO organoids grew slower than WT organoids in medium containing growth-promoting ligands (Fig. 2C). Further analyses of organoid cells revealed that the rate of proliferation was moderately, but significantly decreased in *Apc*-KO organoids (Fig. 3B). The biological importance of this phenomenon is unclear, but it has been shown that an optimal level of Wnt/β-catenin signaling is important for promoting cell proliferation, and excessive activation rather inhibits proliferation of intestinal epithelial cells^33^. Therefore, we think that it is quite possible that excessive activation of Wnt/β-catenin signaling delays organoid growth and cell cycle progression in *Apc*-KO organoids in the presence of WRN-CM. Actually, qRT-PCR analyses showed that *Axin2* expression was much higher in *Apc*-KO organoids than in WT organoids in the presence of WRN-CM (Fig. 1E). This idea is consistent with “just-right signaling model” that a just-right level of Wnt/β-catenin signaling is important for tumor development^34,35^. Alternatively, APC has been reported to play an important role not only in suppressing Wnt/β-catenin signaling, but also in regulating cell morphology and division. Related to this phenomenon, it is noteworthy that APC binds to the plus ends of microtubules and plays an important role in chromosome alignment and segregation during mitosis^36,37^. Therefore, *Apc*-KO may interfere with the proper progression of mitosis, thereby delaying the speed of cell proliferation.

The cross-sectional area of cells in *Apc*-KO organoids was significantly larger than that in WT organoids (Fig. 4), suggesting the increase in cell size in *Apc*-KO organoids. Cell size regulation is an important issue in cell biology and the importance of mechanistic target of rapamycin complex 1 (mTORC1) signaling in its regulation has been well established^38,39^. Constitutive activation of mTORC1 signaling results in an increase in cell size by promoting protein synthesis and cell growth across various species. Several reports have shown the functional link between APC and mTORC1: APC can suppress mTORC1 activity by stimulating GSK3^40^, and *Apc* mutations result in the strong activation of mTORC1 in vivo in both mice and zebrafish^41^. Moreover, treatment of *Apc*-heterozygous mutant mice or *Apc*-deleted mice with mTORC1 inhibitors effectively suppressed polyp formation/growth and reduced mortality^42–44^, implying the crucial importance of this functional connection. Therefore, the increase in cross-sectional area observed in *Apc*-KO colon organoids might be attributable to the constitutive activation of the mTORC1-signaling pathway.

As mentioned above, *Apc*-KO generally suppressed differentiation into major cells that constitute the colon epithelia (Fig. 5). In contrast, we observed the presence of lysozyme-positive cells, specifically in *Apc*-KO organoids, independent of ligand stimulation (Fig. 6C). Such lysozyme-positive cells are normally seen in the small intestine, but not in the colon or in the colon-derived organoids (Fig. 6A, B), and are known to represent Paneth cells, which constitute the stem cell niche. Therefore, the ectopic emergence of lysozyme-positive cells in *Apc*-KO colon organoids is a notable event. Several reports have shown the presence of similar lysozyme-positive cells in human colon cancer tissues^30–32^; chemical-induced in vivo acute deletion of *Apc* genes in the mouse colon also resulted in the emergence of lysozyme-positive cells^31,32^. In these mice, deletion of exon 14 caused a frameshift mutation at the beginning of exon 15^11,45^. Both these mice and *Apc*-KO organoids commonly lack the region encoded by exon 15, and this region is supposed to be needed for suppression of ectopic cell differentiation. As β-catenin activation has been reported to increase the number of lysozyme-positive cells in intestinal epithelia^31^, excessive activation of WNT/β-catenin signaling caused by deletion of exon 15 might lead to ectopic cell differentiation. Our study not only confirmed the important role of APC in suppressing the differentiation into lysozyme-positive cells in the colon, but also clearly demonstrated that differentiation can occur autonomously in the epithelial cells without the help of other types of cells around the epithelia. Considering the stem cell-maintaining role of the Paneth cells in the small intestine, it is probable that the ectopic differentiation of lysozyme-positive cells induced by *Apc*-KO significantly contributes to cancer development. The colon organoids we used in this study would be an ideal tool to further investigate the molecular mechanism and biological importance of ectopic differentiation.

## Methods

### Animal experiments

4-month-old female mice of C57BL/6J strain were used for organoid culture. 8-month-old male mice were used for immunofluorescence staining experiments. All animal experiments were conducted in accordance with the ARRIVE guidelines and the guidelines for proper conduct of animal experiments issued by the Science Council of Japan. All experimental protocols were approved by the institutional review board of Osaka University.

### Reagents

We used the following primary antibodies: anti-APC (Cell Signaling Technology, 2504, 1:1000); anti-β-catenin (BD transduction laboratories, 610154, 1:2000); anti-β-actin (Sigma-Aldrich, A-2228, 1:2000); anti-E-cadherin (BD Biosciences, 61082, 1:100); anti-Ki67 (Cell Signaling Technology, 12202, 1:50); anti-cleaved caspase3 (Cell Signaling Technology, 9664, 1:50); anti-Mucin-2 (Santa Cruz Biotechnology, sc-15334, 1:50); anti-chromogranin A (Abcam, ab15160, 1:50); and anti-lysozyme antibodies (Thermo Fisher Scientific, RB372-A1, 1:50). We used the following secondary antibodies: Alexa-Fluor-488-conjugated anti-rabbit and anti-mouse-IgG antibodies (Invitrogen, 1:200) for immunofluorescence staining and alkaline phosphatase-conjugated anti-mouse-IgG antibody (Promega, 1:10,000) for immunoblotting analyses. We used the following reagents: advanced DMEM/F-12 (Ad-DF; Invitrogen); B-27 supplement (Gibco), BrdU Labeling and Detection Kit II (Roche); Cell Recovery Solution (Corning); dibenzazepine (DBZ; CAYMAN); epidermal growth factor (EGF; Invitrogen); fetal bovine serum (FBS; Biowest); GlutaMAX-I (Invitrogen); L-161982 (CAYMAN); Lipofectamine 2000 (Invitrogen); Luna Universal One-Step RT-qPCR Kit (New England Biolabs); Matrigel (growth factor reduced, phenol red free; BD Biosciences); N-2 supplement (Gibco); Noggin (Peprotech); Proteinase K (Wako); rhodamine-labeled phalloidin (Wako); RNAiso Plus (Takara); TrypLE Express (Invitrogen); and Y-27632 (Wako).

### Organoid culture

Colon-derived organoids were cultured as described previously^17^. To prepare WRN-CM, L-WRN cells (CRL-3276; ATCC) were cultured in Ad-DF supplemented with 20% FBS, 1 × GlutaMAX-I, 100 U/mL penicillin, and 100 μg/mL streptomycin (primary culture medium; PCM). The culture medium was collected every day for 8 days. It was diluted twice with PCM (50% WRN-CM medium) and used for culture. The organoids were passaged as follows: Matrigel containing the organoids was washed with phosphate buffered saline (PBS) containing 0.5 mM EDTA and was treated with TrypLE Express for 5 min at 37 °C. Dissociated cells were washed with Ad-DF and then resuspended in Matrigel. The organoids were passaged every 3 days for two weeks and then used for the experiments. Two WT cell lines (WT#1 and WT#2) were established from two different mice.

Culture of small intestine-derived organoids was performed as described previously^12^, with some modifications. Briefly, the proximal quarter of a small intestine was minced into 5-mm pieces and then incubated with PBS containing 20 mM EDTA for 2 h at 4 °C. Crypts were isolated from the tissue pieces by vigorous pipetting in PBS containing 10% FBS. After removal of the pieces, the supernatants containing the crypts were centrifuged, and then the pellets were suspended in Matrigel and cultured in ENR medium (Ad-DF supplemented with 1 × GlutaMAX-I, 10 mM Hepes, 100 U/mL penicillin, 100 μg/mL streptomycin, 1 × N-2 supplement, 1 × B-27 supplement, 1.25 mM N-Acetylcysteine, 50 ng/mL EGF, 100 ng/mL Noggin, 10% R-spondin1 CM^17^).

### Generation of *Apc*-KO organoids

*Apc*-KO organoids were generated using the CRISPR/Cas9 system as follows. *Apc*-specific sgRNA oligos were inserted into the px330 expression vector (Addgene). The target sequence was 5′-CAAGCACAAAATGATTGCCA-3′^14^. Organoids grown in 50% WRN-CM medium were washed with PBS containing 0.5 mM EDTA, and then incubated with TrypLE Express for 5 min at 37 °C. Trypsinized cells were dissociated into single cells by vigorous pipetting, resuspended in 50% WRN-CM medium containing 10 μM Y-27632, and then plated in 24-well plates. The targeting plasmid was introduced into single cells using the Lipofectamine 2000 reagent. After adding the transfection reagent, the plate was centrifuged at 600×*g* for 1 h at room temperature, and then incubated for 4 h at 37 °C. The transfected cells were collected, resuspended in Matrigel, and cultured in 50% WRN-CM medium containing 10 µM Y-27632 for 3 days. To select the *Apc*-KO cells, the medium was replaced with PCM, and the cells were cultured for 9 more days. When the cells formed organoids, Matrigel was depolymerized using Cell Recovery Solution for 30 min at 4 °C. Each single cell-derived organoid was picked up, incubated with TrypLE Express, suspended in Matrigel, and then cultured in PCM. After 3 days, Matrigel was removed using Cell Recovery Solution. The organoids were collected, washed twice with PBS, and then about 100 organoids were suspended in 50 μL of DNA extraction buffer (10 mM Tris–HCl pH 8.0, 50 mM KCl, 1.5 mM MgCl_2_, 0.1% gelatin, 0.45% Tween 20, and 0.4% NP-40) containing 0.1 mg/mL Proteinase K. The samples were incubated for 3 h at 56 °C, boiled for 10 min at 95 °C, and then centrifuged at 13,000×*g* for 1 min. The resulting supernatants were used as a template for PCR amplification of the genomic region spanning the target site using the following primer sets: 5′-GCACTTGAAATCTCACAGCT-3′ and 5′-TGAGCATCTAGCTCAGCTTC-3′. The PCR products were cloned into T-vector pMD20 (Takara), and then 8 independent plasmids were sequenced for each sample. We confirmed the existence of two types of frameshift mutant alleles at the targeted locus in each of two independent organoid cell clones, *Apc*-KO#1 and *Apc*-KO#2 (Fig. 1A). As these *Apc*-KO organoid clones were single cell-derived, two types of mutant alleles were supposed to have occurred in a single cell. Indeed, we did not detect three or more different types of *Apc* alleles in each *Apc*-KO clone (Fig. 1A). We mainly analyzed *Apc*-KO#2 organoids, in which both types of mutant alleles were detected several times, in Figs. 3, 4, 5 and 6.

### qRT-PCR

Matrigel was removed using Cell Recovery Solution, as described above. Organoids were collected and total RNA was isolated using RNAiso Plus (Takara) according to the manufacturer’s instructions. Extracted RNA was used as a template for reverse transcription and qRT-PCR using Luna Universal One-Step RT-qPCR Kit (New England Biolabs). Primer sets: *Axin2*^16^, 5′-GCAGCTCAGCAAAAAGGGAAAT-3′ and 5′-TACATGGGGAGCACTGTCTCGT-3′; *Gapdh*, 5′-CTCTTCCACCTTCGATGCC-3′ and 5′-GGGAGATGCTCAGTGTTGG-3′.

### Immunostaining

To stain the organoids, Matrigel was removed using Cell Recovery Solution, as described above. Organoids were collected, fixed with PBS containing 4% paraformaldehyde (PFA) for 10 min, and then permeabilized with PBS containing 0.5% Triton X-100 for 5 min. After blocking with PBS containing 3% FBS and 10% BSA (blocking buffer) for 1 h at room temperature, the organoids were incubated with the primary antibodies diluted in blocking buffer for 12 h at 4 °C, and then incubated with the fluorescence-labeled secondary antibodies for 1 h at room temperature. The organoids were suspended in blocking buffer and mounted on glass slides. For staining tissue sections, the freshly dissected small intestine and colon were fixed with PBS containing 4% PFA for 30 min, embedded in OCT compound, frozen using liquid nitrogen, and then sectioned into 20-μm-thick slices. The sections mounted on glass slides were fixed with PBS containing 4% PFA for 10 min and blocked with blocking buffer for 1 h at room temperature. After incubation with anti-lysozyme antibody overnight at 4 °C, the specimens were incubated with fluorescence-labeled secondary antibodies for 45 min at room temperature. Rhodamine-labeled phalloidin and DAPI were used to counterstain F-actin and DNA, respectively. The specimens were examined using a confocal laser scanning microscope (FluoView FV1000; Olympus).

### BrdU staining

BrdU Labeling & Detection Kit II (Roche) was used to label and detect S-phase cells in organoids. Organoids were incubated with 25 μM BrdU for 8 h. They were recovered from Matrigel using Cell Recovery Solution, and then fixed using 70% ethanol containing 15 mM glycine for 20 min at − 30 °C. Fixed organoids were incubated with anti-BrdU antibodies for 2 h at 37 °C, and then incubated with the fluorescence-labeled secondary antibodies for 30 min at room temperature. The specimens were prepared as described above and observed using a confocal laser scanning microscope.

### Alkaline phosphatase staining

Organoids were fixed using PBS containing 4% PFA for 10 min at room temperature, and then recovered from Matrigel using Cell Recovery Solution. When treated with L-161982, fixed organoids were embedded in OCT compound, frozen using liquid nitrogen, and then sectioned into 20-μm-thick slices. Fixed organoids and sections were incubated with 0.19 mg/mL 5-bromo-4-chloro-3-indolyl phosphate and 0.25 mg/mL nitro blue tetrazolium chloride in buffer (100 mM Tris–HCl pH9.5, 100 mM NaCl, 5 mM MgCl_2_) at room temperature for 10 and 90 min, respectively. The organoids and sections were washed with PBS and observed under a stereo microscope (SZX16; Olympus) and an upright microscope (BX41; Olympus), respectively.

### Statistical analysis

Statistical analyses were performed using Prism 6 (GraphPad). Tukey’s and Bonferroni’s multiple comparison tests were used after one-way analysis of variance to calculate *P* values in Figs. 1E and 4B, respectively. Before performing these statistical tests, we evaluated the distribution of the data by calculating their variance using Prism 6, and verified that the variance was similar among the groups. Dunn’s multiple comparison test was used after the Kruskal–Wallis test to calculate *P* values in Figs. 2C, 3A–C, 4C, E, 5A, B.

## Supplementary Information

Supplementary Figures.

## Data Availability

The datasets generated and/or analyzed during the current study are available from the corresponding author on reasonable request.
