# Supplementary material for: Role of adenomatous polyposis coli in proliferation and differentiation of colon epithelial cells in organoid culture
Source: Sci Rep. 2021 Feb 17;11:3980. doi: 10.1038/s41598-021-83590-6 (PMC7889860; doi:10.1038/s41598-021-83590-6)
Supplement: Supplementary file 1 — Supplementary Figures. [file 41598_2021_83590_MOESM1_ESM.pdf]

Supplementary Information for:

Role of adenomatous polyposis coli in proliferation and differentiation of colon epithelial cells in organoid culture

Daisuke Yamazaki*, Osamu Hashizume, Shiho Taniguchi, Yosuke Funato, and Hiroaki Miki*

Department of Cellular Regulation, Research Institute for Microbial Diseases, Osaka University, Suita, Osaka 565-0871, Japan.

*corresponding authors

e-mail: dayama@biken.osaka-u.ac.jp; hmiki@biken.osaka-u.ac.jp

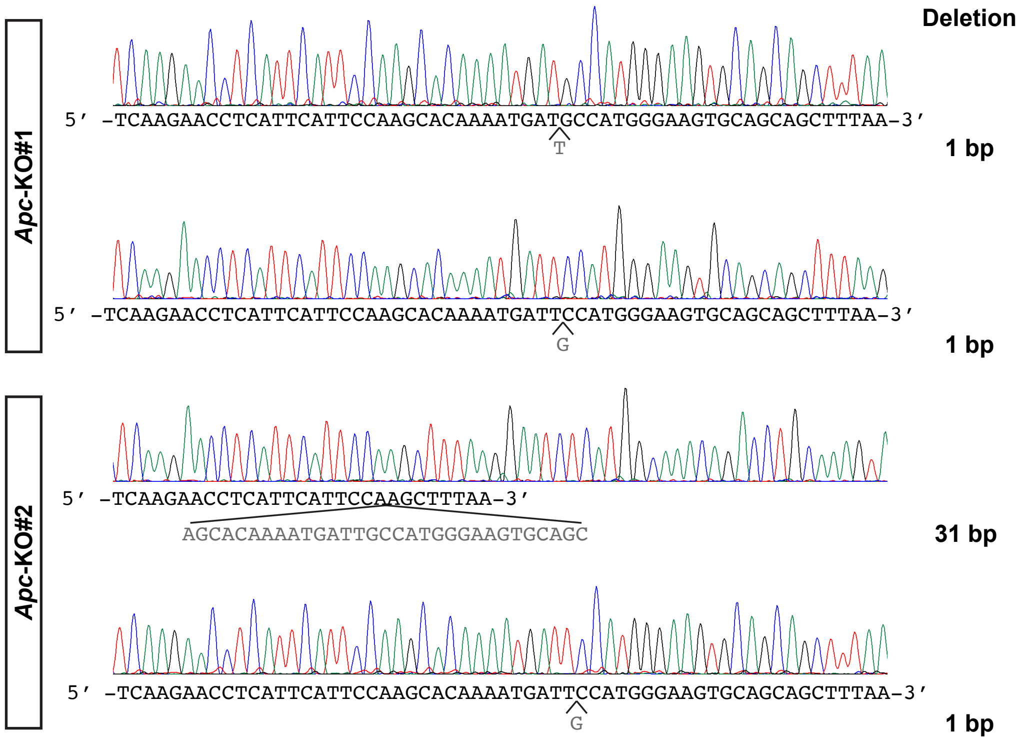

Supplementary Figure S1. Sequencing chromatograms referring to Fig. 1A.

The deleted nucleotides are shown below each sequence.

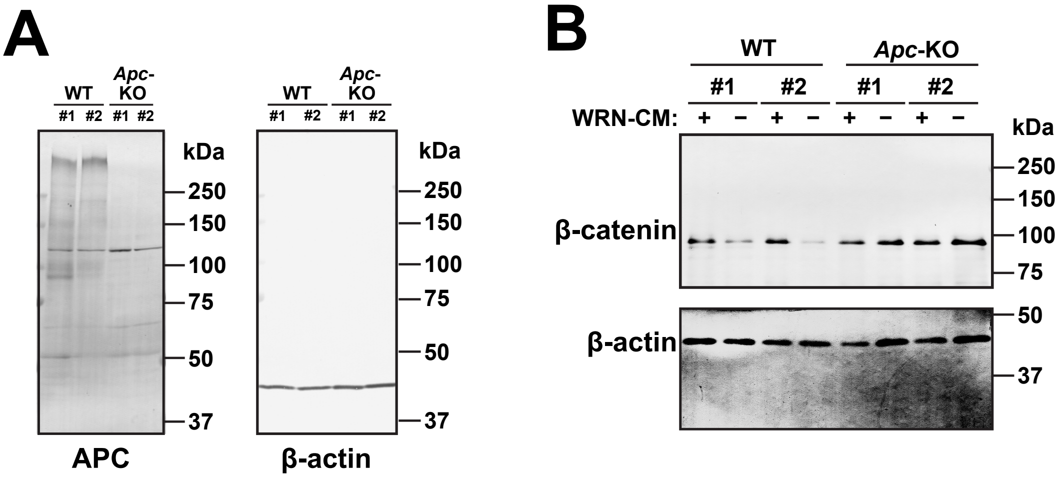

Supplementary Figure S2. Full-length blots referring to Figs. 1C and 1D. (A) Raw data from Fig. 1C. The membranes were incubated with anti-APC and anti-β-actin antibodies, respectively. (B) Raw data from Fig. 1D. The membrane was cut into two parts and the upper and lower parts were incubated with anti-β-catenin and anti-β-actin antibodies, respectively.

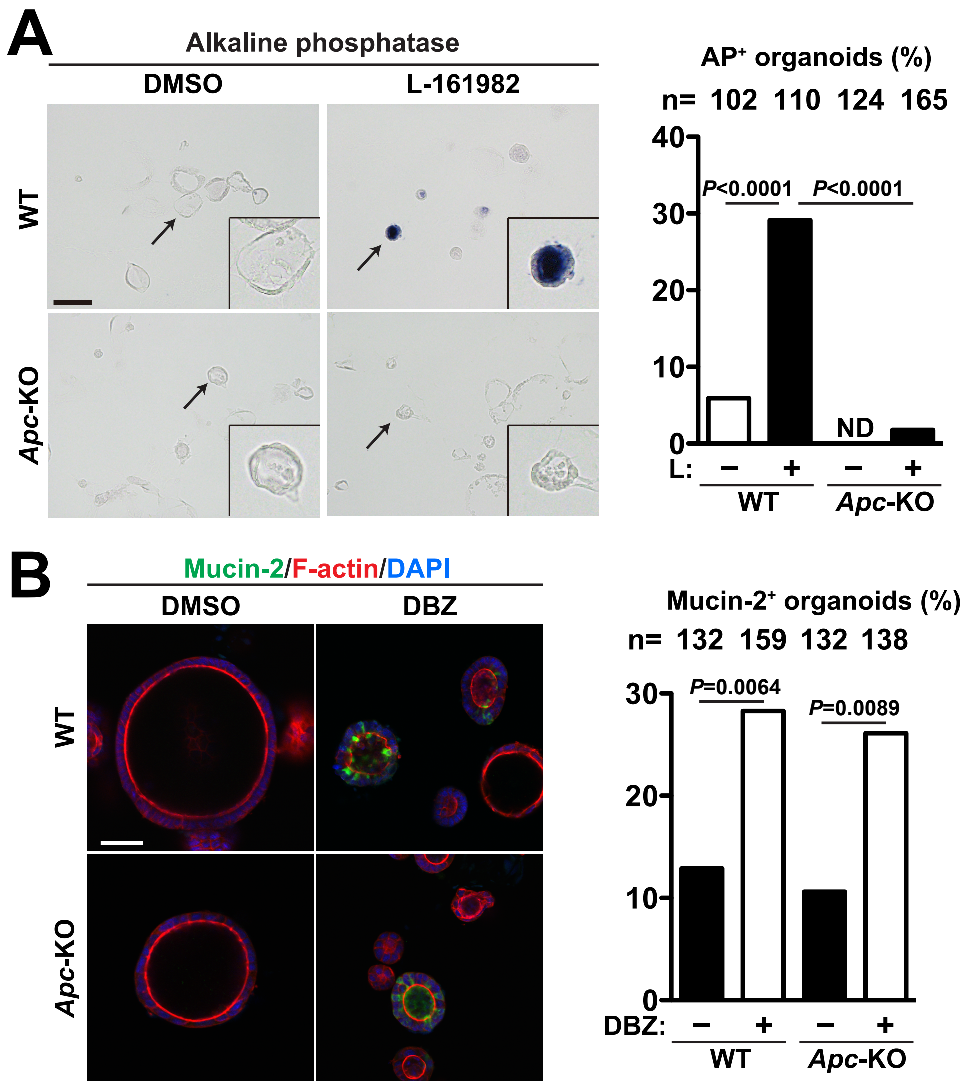

Supplementary Figure S3. Cell differentiation in *Apc*-KO organoids.
(A) Organoids formed in the presence of WRN-CM were cultured in the absence of WRN-CM with or without 20 µM L-161982 for 30 more hours. Sections of the organoids were subjected to alkaline phosphatase staining. The organoids indicated by arrows are enlarged in the insets. The ratio of organoids containing at least one alkaline phosphatase-positive cell to total organoids was quantified for each group. The total number of organoids analyzed is shown above each bar. ND, not detected. Dunn' s multiple comparison test was used after Kruskal-Wallis test to calculate *P* values. Scale bar, 100 µm. (B) Organoids were passaged and then cultured in the presence of WRN-CM with or without 10 µM DBZ for 3 days. The organoids were fixed and then stained using anti-Mucin-2 antibody (green). Counter stain, DAPI for DNA (blue) and phalloidin for F-actin (red). The ratio of organoids containing at least one Mucin-2-positive cell to total organoids was quantified for each group. The total number of organoids analyzed is shown above each bar. Dunn' s multiple comparison test was used after Kruskal-Wallis test to calculate *P* values. Scale bar, 50 µm.
